# The era of high-quality chemical probes

**DOI:** 10.1039/d2md00291d

**Published:** 2022-11-28

**Authors:** Marco P. Licciardello, Paul Workman

**Affiliations:** a Centre for Cancer Drug Discovery, Division of Cancer Therapeutics, The Institute of Cancer Research London UK paul.workman@icr.ac.uk; b The Chemical Probes Portal UK

## Abstract

Small-molecule chemical probes are among the most important tools to study the function of proteins in cells and organisms. Regrettably, the use of weak and non-selective small molecules has generated an abundance of erroneous conclusions in the scientific literature. More recently, minimal criteria have been outlined for investigational compounds, encouraging the selection and use of high-quality chemical probes. Here, we briefly recall the milestones and key initiatives that have paved the way to this new era, illustrate examples of recent high-quality chemical probes and provide our perspective on future challenges and developments.

## Abstract 2

A brief history of milestones leading to selection and use guidelines for high-quality chemical probes. We highlight their importance to study protein function and illustrate examples of recently developed probes acting through different modalities.

## Graphical abstract

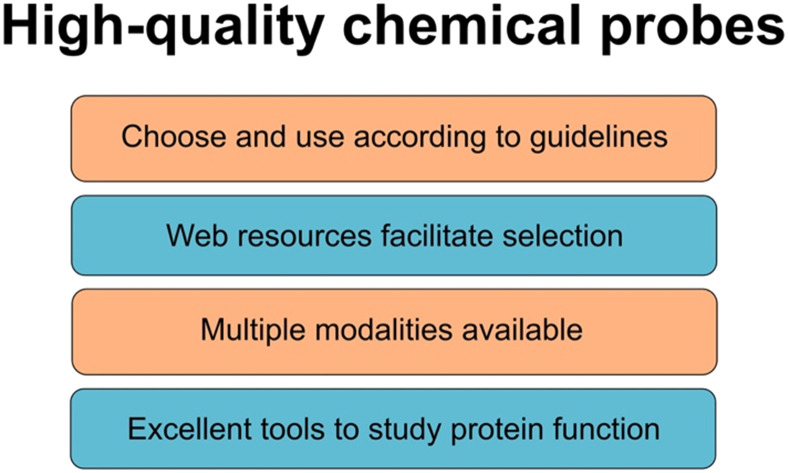

## A brief history of chemical probes

A detailed understanding of the function of every human protein remains one of the greatest challenges in biology. The sequencing of the human genome has revealed a catalogue of roughly 20 000 protein-coding genes and studies performed over the past two decades have linked genetic alterations in some of these genes to diseases (https://www.omim.org/) including cancer^1^ as well as neurodegenerative^2^ and autoimmune disorders.^3^ However, the function of the vast majority of human proteins remains unknown or poorly understood.^4,5^ Importantly, there is clear evidence that protein annotation is stimulated by the discovery and availability of high-quality chemical probes.^6^

Chemical probes are highly characterized small molecules that can be used to investigate the biology of specific proteins in biochemical and cellular assays as well as in more complex *in vivo* settings.^7,8^ Commonly, chemical probes modulate the biological function of a protein through binding to orthosteric or allosteric pockets in the three-dimensional structure of their targets. Traditionally, most of these are inhibitors, antagonists or agonists. More recently, small molecules inducing target protein degradation, also referred to as protein degraders, have attracted much interest. Molecular glues are bifunctional molecules recruiting E3 ubiquitin ligases in close proximity to specific target proteins. PROteolysis TArgeting Chimeras (PROTACs) act in a similar way and expand the target space of protein degraders by linking an E3 ligase recruiter to a small molecule binding a protein of interest.^9–11^ The ternary complex induced by protein degraders leads to target ubiquitination and proteasome-dependent degradation. Moreover, the tripartite interaction can endow degraders with striking selectivity even when the protein target-binding arm of the molecule exhibits some level of off-target activity. In contrast to gene knockout by means of biological tools, PROTACs and molecular glues lead to a concentration-dependent degradation of their target that usually occurs within hours. Therefore, degraders provide greater control when investigating the overall function of a protein, including any scaffold-dependent activity. Proteins with a well-defined small-molecule-binding pocket represent only a fraction of therapeutically relevant targets. Indeed, many proteins exert their function solely by interacting with other macromolecules inside the cell and do not bind naturally occurring small molecules, such as enzyme substrates or other ligands, under physiological conditions. Although PROTACs and molecular glues still need to physically engage with their targets, they do not require tight binding to induce protein degradation. Therefore, degraders provide a route to target proteins with less well-defined small-molecule-binding clefts, many of which are considered ‘undruggable’ by conventional means. Protein degraders are also now finding their way to patients with at least 15 bifunctional molecules in clinical trials by the end of 2021.^12^

PROTACs and molecular glues are not the only tools available to target ‘undruggable’ proteins. Although protein–protein interactions (PPIs) mediated by large surface areas have long been considered hard to target, the molecular interactions across these surfaces are not evenly distributed, but in many cases occur at specific regions termed ‘hot spots’. As we illustrate with one of the examples below, it is possible to design chemical probes targeting these ‘hot spots’ to interfere with PPIs spanning relatively large surface areas.^13,14^

**Fig. 1:**
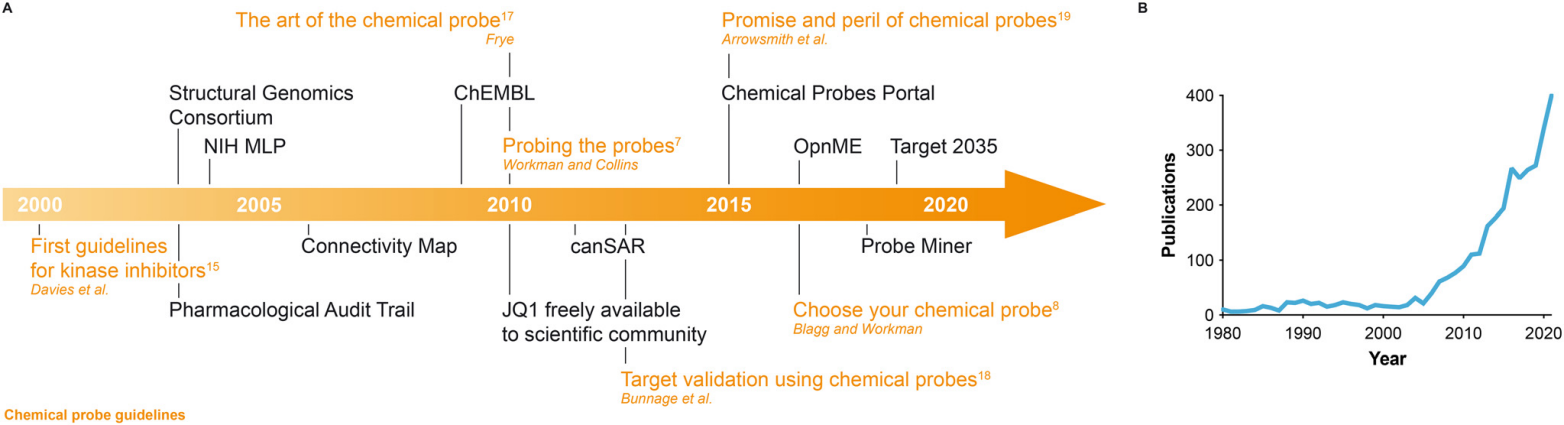
(A) Timeline of key milestones, initiatives and reviews with guidelines leading to the era of high-quality chemical probes. For chemical probes guidelines, the corresponding reference in the text is indicated. (B) Number of PubMed entries per year containing the words ‘chemical probe(s)’ in the title or abstract.

Synthetic small molecules and natural products have long been used to explore the function of proteins in cell biology and pharmacology,^7^ but weak and non-selective compounds have led to confounding results in the past. The systematic evaluation of the selectivity of small-molecule tool compounds was initiated by Cohen and colleagues who published in the year 2000 the investigation of commonly used kinase inhibitors that were frequently assumed to be specific for a single target protein, but which actually inhibit – at times even more potently – one or more additional kinases. These observations led to the first guidelines for the selection of high-quality small molecule inhibitors to study the function of protein kinases^15,16^ (Fig. 1A). Subsequently, the chemical biology community has produced minimal criteria or ‘fitness factors’ to define high-quality small-molecule chemical probes suitable for the investigation of protein function.^7,8,17–19^ These criteria aim to aid the selection and best practice use for biologists, including those who are less familiar than chemical biologists and drug discovery scientists with the potential faults and limitations of compounds claimed to be chemical probes.

According to consensus criteria, chemical probes must be potent (IC_50_ or *K*_d_ < 100 nM in biochemical assays, EC_50_ < 1 μM in cellular assays) and selective (selectivity >30-fold within the protein target family, with extensive profiling of off-targets also outside the protein target family). In cellular and organismal models, strong evidence of on-target engagement and modulation should be provided in accordance with the Pharmacological Audit Trail concept.^20,21^ For chemical probes to be used in animals, information should be provided on species used, dose, administration route, and vehicle as well as on suitable pharmacokinetic data, including peak plasma concentration and time to reach it, elimination half-life and clearance; in addition, data on protein-bound fraction and unbound (free) concentration of the compound in plasma and tissues should be provided.^22,23^ Chemical probes must not be highly reactive promiscuous molecules^24^ and care should be taken with compounds that behave as nuisance compounds in relevant bioassays.^25^ Nonspecific electrophiles, redox cyclers, chelators and colloidal aggregators that modulate biological targets promiscuously through undesirable mechanisms of action should be avoided. Care should also be taken to avoid compounds that do not modulate bioactivity but rather generate artifacts solely by interfering with assay readouts.

While the design and optimization of high-quality chemical probes should avoid these most egregious undesirable behaviors, attention should also be paid to compounds that modulate the desired target but also affect even a relatively small number of off-targets within or outside the same protein family. Additionally, inactive analogues, which are usually assumed to bind only the off-targets of the corresponding active small molecule, should be used alongside high-quality chemical probes to support the association between on-target engagement/modulation and a corresponding phenotype. However, the off-target spectrum of inactive analogues should be investigated in detail as even minor structural changes can lead to non-overlapping off-target profiles between active probes and their corresponding inactive analogue.^26^ Finally, best practice also requires the use of a structurally distinct high-quality chemical probe targeting the same protein whenever possible.^8,19^

The last two decades have seen a number of key initiatives aimed at developing new small molecules targeting especially disease-relevant proteins or providing resources to select the best available chemical probes (Fig. 1A). Between 2004 and 2008, the US National Institute of Health (NIH) launched the Molecular Libraries Program (MLP), coordinated 10 high-throughput screening centres and released 64 chemical probes. However, a later expert assessment concluded that 25% of these molecules inspired little confidence as genuine probes, the citation rate of these compounds has remained low and their accessibility through commercial vendors is limited to a restricted number.^27,28^ It can be argued that chemical probes only reach their full potential when unencumbered and widely disseminated, as exemplified by the seminal case of JQ1, a BET family bromodomain inhibitor identified in a collaboration between the Dana-Farber Cancer Institute and the Structural Genomics Consortium (SGC) Chemical Probes Collection (https://www.thesgc.org/chemical-probes).^29^ Unencumbered access to JQ1 stimulated a great amount of original research on bromodomain-containing proteins,^30,31^ which had been relatively unexplored previously. JQ1 inhibits all members of the BET family and is regarded as a good pan-BET chemical probe. Note that compounds acting as dual inhibitors of the BET family member BRD4 and protein kinases have been described.^32^ Following JQ1, the SGC, also in collaboration with seven pharmaceutical companies, has identified and released more than 100 chemical probes targeting bromodomain-containing^33^ and other epigenetic proteins,^34^ protein kinases,^35^ G protein-coupled receptors (GPCRs)^36^ and additional protein classes. Similarly, Boehringer Ingelheim has launched the OpnMe portal (https://opnme.com) to provide in-house-developed high-quality small molecules freely or *via* scientific research submissions.^37^ Alternatively, chemical probes can be purchased from different suppliers, although we note that in these instances non-critical advice can prevail over critical recommendations that are essential to choose the best available tool compound to investigate a specific target.

Selecting the best chemical probe available for biological studies is not a trivial task, especially when the target of interest is not covered by the probe collections mentioned above. Information may be provided across a diverse range of sources and biomedical researchers unfamiliar with chemical probes often rely on citation rates or search engine results. However, these are often biased towards older compounds that are either of very poor quality *per se* but were nevertheless frequently and erroneously used in the past, or that are early pathfinder/tool molecules that were useful at one time but have been superseded by higher-quality, usually more selective chemical probes. The chemical probe resources available to help researchers select the best possible chemical probes have been reviewed comprehensively by Antolin and colleagues.^38^ The Chemical Probes Portal (https://www.chemicalprobes.org) was launched in 2015 (ref. 19, 39 and 40) and currently lists 771 small molecules (including ‘historical compounds’ – small molecules extensively used in the literature but which do not meet the criteria of high-quality chemical probes) targeting over 400 different proteins and around 100 protein families.^41^ Either chemical probes or target proteins can be easily searched by name. Compounds listed on the portal are reviewed and scored by the large Scientific Expert Review Panel (SERP) using a 4-star grading system. The page for each probe contains useful information, references and comments by SERP reviewers on best use, including the appropriate concentration range or dose and any limitations to be considered. The chemical probe and target space covered by the Chemical Probes Portal is limited by the pace of expert curation and, in addition, some researchers may prefer a more comprehensive and objective database from which to select the best available small molecule to investigate their target. Launched in 2018, the Probe Miner platform^42^ (https://probeminer.icr.ac.uk) provides a statistically-based ranking derived from mining bioactivity data on >1.8 million small molecules and >2200 human targets compiled mainly from the extensive medicinal chemistry literature *via* ChEMBL and collected in the canSAR knowledge base^43^ (https://cansarblack.icr.ac.uk/). The data are used to rank small molecules according to six criteria based on the minimal criteria for high-quality chemical probes that we have outlined above.^42^ This allows the research community to retrieve the best small molecules available for a protein of interest even when the target is not covered by the Chemical Probes Portal or other chemical probe databases. However, bioactive molecules may not satisfy all minimal criteria for high-quality chemical probes. Moreover, information in some public databases can be limited or inconsistently reported. Researchers should acknowledge these shortcomings when retrieving information from databases that are not curated by experts and critically assess the suitability of the chemical probe selected. We recommend the use of the statistically-based Probe Miner alongside the expert review-based Chemical Probes Portal as complementary resources. The portal is recommended as a user-friendly resource for those who are not experts in chemical probes, chemical biology, or drug discovery.

We are witnessing a growing awareness of the importance of using high-quality chemical tools when investigating protein function, as suggested by the increasing number of potent and selective small molecules being developed, published and disseminated and also by the growing number of PubMed entries containing the words ‘chemical probe(s)’ in the title or abstract (Fig. 1B). However, high-quality small molecules are not available for every human protein, limiting functional studies in those instances to the use of biological tools. Antibodies can be excellent reagents to study protein function but the availability of high-quality, specific antibodies is restricted to a limited number of proteins and a high degree of rigour is required in their selection.^44^ Moreover, antibodies are usually restricted to extracellular proteins and lack peroral and brain bioavailability. Genetic tools can be extremely useful but some, such as small interfering RNAs (siRNAs), may be less specific than high-quality chemical probes and often do not provide as much control over the kinetics of target modulation.^8,45^ Nevertheless, the more recent advent of clustered regularly interspaced short palindromic repeat (CRISPR)-based biological tools has provided greater levels of specificity.^46^ The CRISPR-mediated genetic removal of a target can be used to validate the context-dependent essentiality of a specific protein, discern on-target from off-target effects of small molecules and evaluate their pharmacological potential.^47,48^ These are crucial aspects that need to be addressed as early as possible in drug discovery, in order to minimize failure at later stages. Indeed, it has recently been shown for several anticancer drugs undergoing clinical trials that they actually exert their biological activity through off-target mechanisms.^47^ Using CRISPR, Sheltzer and colleagues demonstrated that upon depletion of the claimed main target (*e.g.* HDAC6, PAK4, PBK), these inhibitors continued to show target-independent cell killing. Moreover, depletion of the claimed targets by CRISPR did not impair cellular fitness arguing against the essentiality of these proteins in cancer, despite the fact the targets had been validated earlier by RNAi and claimed chemical probes. Evaluation of putative chemical probes in CRISPR knockout cell lines can now be recommended as a further check for on-target *versus* off-target activity. Genetic technology can also be used to specifically mutate protein residues to modulate binding to a chemical probe. This can help validate target engagement and can corroborate the association with downstream biomarkers in cells. We thus encourage the use of suitable biological tools alongside high-quality chemical probes when studying protein function.

These days, chemical probes are commonly developed following a target-based approach where biochemical screens are conducted and 3D protein structural information is used to generate a potent and selective small molecule that interferes with the function of a specific protein. However, chemical probes may also arise from phenotypic or pathway screens, which require downstream target deconvolution and elucidation of the mechanism of action. Target identification is commonly carried out using quantitative mass spectrometry-based proteomic technologies.^49^ Unraveling the precise mechanism of action of small molecules can be particularly arduous when the target is a relatively unknown protein acting as a hub in the molecular network of the cell. Nevertheless, it is possible to generate hypotheses by interrogating databases of genetic and chemical cellular perturbations such as those generated within the Library of Integrated Network-Based Cellular Signatures (LINCS), a consortium funded by the NIH.^50^ Among the different LINCS centres, the L1000 transcriptomics platform at the Broad Institute^51^ has collected ‘reduced’ transcription profiles (based on around 1000 transcripts) for multiple variations (concentration, time point, cell line) of different perturbagens in cancer cell lines, expanding the original concept of the Connectivity Map.^52^ This and other databases collecting information on the perturbed proteome or epigenome, can be queried with the molecular signature of a compound of interest to identify perturbagens inducing similar profiles and suggesting potential molecular mechanisms of action.

**Fig. 2:**
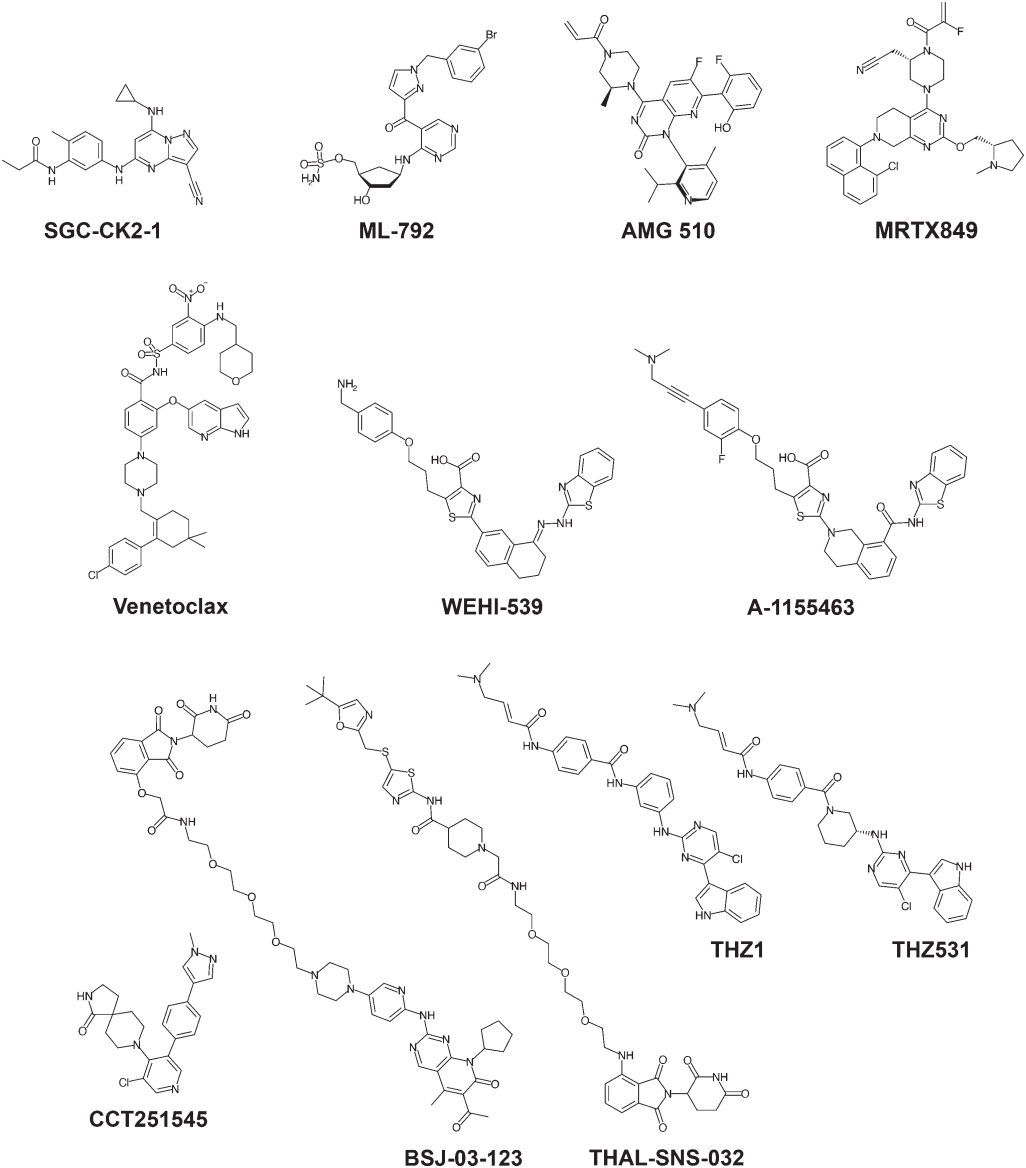
Chemical structures of recent high-quality chemical probes discussed in this article.

As the number of high-quality chemical probes available to the research community increases, so does our knowledge of protein function and human biology. In the following sections, we will illustrate examples and discuss potential applications of recent chemical probes meeting the high-quality criteria outlined above, spanning different protein families and mechanisms of action (Fig. 2).

## Probing the essentiality of CK2

Casein kinase 2 (CK2) is a serine/threonine protein kinase that has long been considered a potential vulnerability in cancer as it has been found to be highly expressed in a range of malignancies and linked to oncogenic mechanisms.^53^ Silmitasertib (CX-4945) is currently the most widely used CK2 inhibitor, has been granted orphan drug status by the US Food and Drug Administration (FDA) in 2017 for the treatment of advanced cholangiocarcinomas (bile duct cancers) and is currently being evaluated in clinical trials for different malignancies. However, silmitasertib has been shown to inhibit kinases other than CK2.^54^ Drug polypharmacology does not necessarily interfere with efficacy or cause toxicity and can even be desirable for therapeutic activity. Nevertheless, compared to drugs, high-quality chemical probes must meet more stringent selectivity criteria, so that the biology of the corresponding target can be investigated with confidence.

SGC-CK2-1, a new high-quality chemical probe for CK2, has been recently developed starting from an earlier series of pyrazolopyrimidine-based compounds.^35,55^ SGC-CK2-1 is exquisitely selective for CK2 in biochemical assays, engages CK2 and reduces the phosphorylation of a validated CK2 substrate in cells. Of note, SGC-CK2-1 fails to modulate the phosphorylation of the same substrate in cells engineered to express a mutant CK2, which cannot bind SGC-CK2-1. Using structural information to confirm target engagement and subsequent effects on downstream substrates in cells is in line with the Pharmacological Audit Trail.^20,21^ This also provides a good example of how chemical and biological tools synergize in corroborating the association between a small molecule, its corresponding target and downstream phenotypes.

In light of this convincing evidence, the present authors support the adoption of SGC-CK2-1 as the new gold standard chemical probe for CK2 in cellular studies. SGC-CK2-1 has already been added to the SGC Chemical Probes Collection (https://www.thesgc.org/chemical-probes/SGC-CK2-1) as well as in the Chemical Probes Portal (https://www.chemicalprobes.org/sgc-ck2-1), albeit with a caveat raised. Alongside SGC-CK2-1, we also encourage the use of its analogue SGC-CK2-1N as an inactive control and the development of an additional, alternative chemotype high-quality chemical probe targeting CK2 to be used in parallel, as recommended by best practice guidelines.

Surprisingly, SGC-CK2-1 showed antiproliferative effects only in a limited number of cancer cell lines within a large panel, thus challenging the broad essentiality of CK2, the mechanism of action of silmitasertib and the general application of CK2 inhibitors as pharmacological treatments in cancer. However, the essentiality of CK2 might be context-dependent and this needs to be further investigated. The use of a very selective high-quality chemical probe alongside biological tools will help elucidate the role of CK2 in malignancy and determine the specific therapeutic potential of CK2 inhibition in cancer. Interestingly, CK2 has been recently implicated in the molecular pathology of other disorders such as amyotrophic lateral sclerosis, Alzheimer's and Parkinson's disease^56^ and also the coronavirus disease 2019 (COVID-19).^57^ These may be alternative therapeutic areas for SGC-CK2-1 and other selective CK2 inhibitors.

## A high-quality chemical probe for SUMO

The small ubiquitin-like modifier (SUMO) is a post-translational modification (PTM) that can be used by cells to modulate the localization, activity, and interactions of target proteins. Recently, there has been a growing interest in the development of SUMOylation inhibitors as this PTM has been implicated in the pathology of Alzheimer's disease and MYC-driven cancer.^58^ However, the biology of SUMOylation has thus far been investigated using mainly biological tools or weak small-molecule inhibitors of the SUMOylation machinery.^59–61^ In the absence of high-quality chemical probes, it is acceptable initially to use such pathfinder compounds especially when biological tools are concomitantly applied to increase confidence in the conclusions.^7^ When this is done, it is important that the limitations of the initial tool compound are clearly revealed. Nevertheless, the application of pathfinder or historical compounds should cease as soon as high-quality chemical probes become available for the same target.

The research community can now rely on ML-792, a potent and selective inhibitor of the SUMOylation machinery.^62,63^ Assisted by the catalytic activity of the SAE1/SAE2 complex of the SUMOylation machinery, ML-792 forms an adduct with SUMO, which prevents transfer of this PTM to the final target. In spite of the similarities between SUMO, NEDD8, ubiquitin and their respective cellular machineries, ML-792 is exquisitely selective for protein SUMOylation and does not inhibit neddylation or ubiquitination in biochemical assays and in cells. Selectivity measurements extended to 366 ATP-using enzymes show no inhibition by ML-792 at a concentration of 1 μM. In cells, ML-792 leads to a concentration-dependent decrease of global protein SUMOylation in as little as 4 hours. Importantly, treatment of cancer cells with ML-792 results in the disruption of nuclear regions rich in SUMOylated substrates and referred to as promyelocytic leukemia nuclear bodies.

SUMOylation has been described as a non-oncogene addiction in NOTCH1-^60^ and MYC-driven cancers.^59,64^ Notably, human cancer cells with high MYC levels show greater sensitivity to ML-792 compared to cells with low MYC levels. Moreover, ML-792 leads to mitotic disruption and chromosome-segregation defects in line with the crucial role played by SUMOylated proteins in mitosis.^65^ Unexpectedly, treatment with ML-792 does not result in considerable perturbation of the transcriptome and proteome of different human cancer cells and changes are dependent on the cellular context. Overall, this suggests that inhibition of SUMOylation can be therapeutically explored and its potential applications will likely be tailored to specific SUMOylation-dependent malignancies.

ML-792 represents a considerable improvement over previous SUMOylation inhibitors and meets the criteria of a high-quality chemical probe. The present authors encourage the adoption of this small molecule for future studies of the biology of SUMOylation and support the development of an inactive analogue as well as a second, structurally distinct, high-quality chemical probe to be used alongside.

## Large areas, small probes

Venetoclax (ABT-199) is a PPI inhibitor designed to hinder the interaction between BCL-2, an antiapoptotic protein overexpressed or amplified in different leukemias, and BH3 domain-containing proapoptotic proteins. Venetoclax was developed starting from the previous, less selective BCL-2 inhibitor navitoclax (ABT-263), which has high affinity for other targets within the same protein family such as BCL-W and BCL-X_L_. The crystal structure of navitoclax bound to BCL-2 was crucial to determine key changes in the architecture of the molecule that would translate into higher selectivity for BCL-2. Venetoclax engages with BCL-2 in biochemical and cellular assays with much greater affinity compared to BCL-W and BCL-X_L_. Moreover, it impairs the fitness of BCL-2-dependent (EC_50_ = 8 nM), but not BCL-X_L_-dependent (EC_50_ = 4260 nM), cells and inhibits the growth of BCL-2-dependent tumors *in vivo*.^66,67^ Venetoclax is recommended by the Chemical Probes Portal where it is highly rated by SERP reviewers for use in cells as well as in model organisms (https://www.chemicalprobes.org/venetoclax). Furthermore, it has been approved for the treatment of chronic lymphocytic, small lymphocytic, and acute myeloid leukemia.

The selectivity of venetoclax has been assessed only within the BCL-2 protein family. Currently, there is no information on interactions between venetoclax and other proteins inside the cell or disruption of ‘off-target PPIs’. However, venetoclax does not kill Bak^−/−^Bax^−/−^ double knockout mouse embryonic fibroblasts, suggesting that it exhibits mainly on-target effects.^66^ Producing an extensive profile of off-targets outside their corresponding target protein family is certainly a more burdensome task with PPI chemical probes as the human interactome is currently predicted to involve 300 000–650 000 binary PPIs.^13,14^ Guidelines specific for these molecules should reflect such differences.

PPI chemical probes targeting other members of the BCL-2 protein family have also been identified. WEHI-539 potently disrupts the interaction between BCL-X_L_ and BH3 domain-containing proteins both potently and selectively.^68^ It is endorsed as a cellular chemical probe by the Chemical Probes Portal for investigating the biology of BCL-X_L_ (https://www.chemicalprobes.org/wehi-539). However, *in vivo* use in model organisms, such as rodents, is not recommended due to its poor physicochemical properties. In contrast, the potent BCL-X_L_ inhibitor A-1155463 has more drug-like features and the measured pharmacokinetic properties as well as demonstration of antitumour activity in mice seem to support *in vivo* applications; however, BCL-W has been reported as a potential off-target^69^ (https://www.chemicalprobes.org/1155463).

## Covalent inhibition of mutant KRAS

The cancer driver KRAS^G12C^ mutation occurs in lung adenocarcinoma, colorectal cancer and also less frequently in other solid tumors. This mutation locks the GTPase KRAS in its active GTP-bound form leading to sustained KRAS signaling and promoting uncontrolled cellular growth and malignancy.^70^ The search for a mutant KRAS inhibitor has long been hindered by its picomolar affinity for GTP and the absence of an alternative well-defined small molecule-binding cleft, which led to the classification of KRAS as ‘undruggable’. However, based on the crystal structure of the oncogenic mutant KRAS^G12C^, which contains a cysteine not present in the wild-type protein, a series of acrylamide-based compounds fitting a previously unknown allosteric pocket adjacent the mutant cysteine of KRAS^G12C^ were identified in 2013.^71^ These small molecules, as well as the later inhibitor ARS-1620 suitable for *in vivo* investigations in mice but with suboptimal potency,^72^ spare the wild-type protein but form a covalent bond with the mutant cysteine of KRAS^G12C^. In contrast to reversible inhibitors, covalent small molecules rely on a non-equilibrium mechanism to achieve their potency and selectivity, which are thus determined by target turnover. The irreversible binding of acrylamide-based compounds favours the inactive GDP-bound form of KRAS^G12C^ and dampens the downstream proliferative signaling. More recently, new covalently-acting acrylamide-based KRAS^G12C^-targeting chemical probes and drugs that show enhanced potency and selectivity have been described.

The structure of AMG 510 (https://www.chemicalprobes.org/amg-510) partly resembles that of ARS-1620 but the additional isopropyl-methylpyridine substituent interacts with the His95 groove of KRAS^G12C^ improving potency by 10-fold in a cell-free biochemical assay.^73,74^ AMG 510 inhibits phosphorylation of ERK, a downstream biomarker of KRAS signaling, and impairs the viability of KRAS^G12C^, but not wild-type KRAS, cancer cell lines (40-fold more potent than ARS-1620). Moreover, AMG 510 inhibits KRAS signaling and tumour growth in xenograft KRAS^G12C^ mouse models. Importantly, cysteine-proteome profiling by mass spectrometry confirms that treatment of cancer cells with AMG 510 for 4 hours at a concentration of 1 μM selectively modifies only the Cys12 peptide from KRAS^G12C^. AMG 510, also known as sotorasib, has been recently granted FDA approval for the treatment of KRAS^G12C^ advanced or metastatic non-small cell lung cancer.

MRTX849 (https://www.chemicalprobes.org/mrtx849) is another covalently-acting high-quality chemical probe identified through structure–activity relationship studies of previous tetrahydropyridopyrimidine-based KRAS^G12C^ inhibitors.^75,76^ Similar to AMG 510, MRTX849 potently and selectively inhibits KRAS^G12C^ signaling and shows antiproliferative effects in cellular as well as KRAS^G12C^-dependent tumour models in mice.^77^ MRTX849 is currently undergoing clinical trials for the treatment of KRAS^G12C^ lung adenocarcinoma and other advanced/metastatic solid tumors. Both AMG 510 and MRTX849 are highly rated at the Chemical Probes Portal and feature among the top ten highlighted compounds added to the portal in 2020 (https://www.chemicalprobes.org/news/2020s-top-probes).

## A growing chemical toolbox for CDKs

Cyclin-dependent kinases (CDKs) are a family of conserved serine/threonine kinases driving fundamental cellular processes such as cell cycle progression, cell division and gene transcription. In cancer, a number of mechanisms leading to increased CDK activity and consequential loss of proliferative controls have been described. Moreover, CDKs have been shown to orchestrate the transcription of genes sustaining malignant transformation.^78^ Because of these observations, many CDKs are considered attractive therapeutic targets. Indeed, the ATP-competitive CDK4/6 inhibitors palbociclib, ribociclib and abemaciclib have been approved by the FDA between 2015–2017 for the treatment of hormone receptor-positive and HER2-negative advanced or metastatic breast cancer.^79–81^ Lately, additional chemical probes targeting different CDKs and based on a range of mechanisms have emerged.

CDK4 and CDK6 regulate the G1-S transition of the cell cycle and elicit homologue-specific functions. However, they share 94% sequence similarity in their ATP-binding pockets and cannot be distinguished by palbociclib. Recently, it has been shown that the strategic installation of a phenoxyacetamide linker conjugated to the E3 ligase recruiter pomalidomide converts palbociclib into BSJ-03-123, a selective PROTAC for CDK6.^82^ BSJ-03-123 shows comparable inhibition of CDK4 and CDK6 in cell-free biochemical assays but leads to the rapid and selective depletion of CDK6 in human cancer cells. Differences in the structure of CDK4 and CDK6 were shown to modulate the formation of the target/degrader/ligase ternary complex in cells resulting in homologue-selective degradation. BSJ-03-123 decreases the phosphorylation of a validated substrate of CDK4/6 and shows antiproliferative effects in CDK6-dependent human acute myeloid leukaemia cells. However, in contrast to the dual CDK4/6 inhibitor palbociclib, BSJ-03-123 fails to impair the proliferation of CDK4-dependent human cancer cell lines, arguing for a negligible effect on the enzymatic activity of CDK4 in cells. Importantly, BSJ-bump, an inactive analogue of BSJ-03-123, fails to degrade CDK6, does not modulate its downstream targets and does not impair the viability of CDK6-dependent cell lines. *In vivo* testing of BSJ-03-123 has not been reported but it is highly rated by the Chemical Probes Portal as a CDK6 PROTAC probe for use in cells *in vitro* (https://www.chemicalprobes.org/bsj-03-123).

CDK9, a component of the positive transcription elongation factor b complex, regulates the transcription of tumor specific genes. THAL-SNS-032 (https://www.chemicalprobes.org/thal-sns-032), a PROTAC generated by the conjugation of the multi-kinase inhibitor SNS-032 to the E3 ligase recruiter thalidomide, inhibits the target kinases of the parental compound in cell-free biochemical assays. However, in contrast to SNS-032, THAL-SNS-032 leads to the selective degradation of CDK9, and partly CDK10, in human cancer cells. The selectivity of THAL-SNS-032 seems to be driven by activity at sub-stoichiometric concentrations but other factors, such as different ubiquitination efficiency, may contribute.^83^ This is a further example of improved chemical probe selectivity obtained by converting a less selective kinase inhibitor into a more specific protein degrader. Treatment with THAL-SNS-032 leads to the rapid degradation of CDK9, decreased phosphorylation of a validated substrate, transcriptional deregulation and apoptosis in human T-cell acute lymphoblastic leukemia (T-ALL) cells, which are particularly sensitive to modulation of transcription. Further assessment of the pharmacokinetic and pharmacodynamic features of this chemical probe will be required to evaluate its potential application in model organisms, such as rodents.

CDK8 and CDK19 comprise the kinase-module of the transcriptional coactivator mediator complex. Potent and selective inhibition of CDK8 and CDK19 can be achieved with the 3,4,5-trisubtituted pyridine-based compound CCT251545, a chemical probe recommended by the Chemical Probes Portal (https://www.chemicalprobes.org/cct251545). The crystal structure of CCT251545 in complex with CDK8 shows that the C-terminal Arg356 inserts into the hinge region to form a cation–pi interaction with the phenyl ring of the inhibitor. This likely explains the high selectivity of CCT251545 for CDK8. CCT251545 reduces the phosphorylation of a validated substrate of CDK8 in human colorectal cancer cells and induces gene expression changes, broadly phenocopying loss of β-catenin in mouse organoids. Indeed, the 3,4,5-trisubtituted pyridine series compounds were originally discovered in a high-throughput phenotypic screen for WNT signaling inhibitors, with target deconvolution by chemoproteomics.^84^ CCT251545 impairs the growth of WNT-dependent tumors in a mouse model but further optimized, highly-selective, orally bioavailable CDK8/19 inhibitors from the 3,4,5-trisubtituted pyridine series, including CCT251921 (ref. 85) – and also those from a different chemotype – show only modest *in vivo* antitumour activity in mouse models and yet exhibit a wide range of multi-organ adverse effects in tolerability studies performed in rat and dog,^86^ which prevented clinical development. Increased super-enhancer activity upon treatment with CDK8/19 inhibitors might explain the absence of a safety window and defines CDK8 and CDK19 as ‘anti-targets’ to be avoided when developing small-molecule kinase inhibitors for therapeutic purposes.

Cysteine-reactive acrylamide-bearing covalent inhibitors have been identified for the transcriptional regulators CDK7, CDK12 and CDK13. THZ1 (https://www.chemicalprobes.org/thz1) binds CDK7 irreversibly through the formation of a covalent bond with Cys312 in close proximity to but outside the canonical ATP-binding pocket of the kinase.^87^ In contrast to its inactive analogue THZ1-R, THZ1 potently inhibits CDK7, leads to decreased phosphorylation of CDK7 substrates and induces anti-proliferative effects in human T-cell acute lymphoblastic leukemia (T-ALL) cells and xenograft mouse models. However, poor solubility and limited stability require further chemical optimizations. Although a useful first-generation probe for CDK7, THZ1 is not ideally selective and the present authors encourage the development and adoption of orthogonal, structurally distinct, CDK7-specific chemical probes to corroborate conclusions.

The closely related CDK12 and CDK13 also harbour a cysteine residue outside but in close proximity to their ATP-binding cleft. The introduction of a piperidine ring in the acrylamide arm of THZ1 shifts selectivity from CDK7 to CDK12/13 and produces the chemical probe THZ531 (ref. 88) (https://www.chemicalprobes.org/thz531). Similar to THZ1, THZ531, but not its inactive analogues THZ531R and THZ532, inhibits the phosphorylation of CDK12/13 substrates, modulates gene expression and induces apoptosis in human T-ALL cells. Although not exquisitely selective for CDK12/13, THZ531 is currently one of the best available chemical probes to study the functions of these two proteins. More recently, a PROTAC chemical probe has been described, based on the multi-kinase inhibitor TAE684 but showing some selectivity for CDK12.^89^ BSJ-4-116 binds to different proteins when assessed in a panel of 468 human kinases at a concentration of 1 μM but degrades mainly CDK12 in T-ALL cells albeit with only a 2-fold selectivity over CDK13, as determined by quantitative proteomics. CDK12-specific structural features allow for the efficient formation of a ternary complex with BSJ-4-116 and an E3 ligase leading to CDK12 proteasome-dependent degradation and sparing CDK13. BSJ-4-116, but not its inactive analogue BSJ-4-116-NC, modulates the phosphorylation of CDK12 substrates and downregulates the expression of genes in the DNA damage response pathway, which are also downregulated upon genetic removal of CDK12. Moreover, BSJ-4-116 impairs the growth of T-ALL cells while BSJ-4-116-NC is 10-fold less potent.

## Towards a chemical probe for every human protein

High-quality chemical probes are now well established as powerful reagents for the functional annotation of proteins and use in target validation for drug discovery, but as highlighted in a recent published discussion^39^ substantial challenges remain for their development, selection and best practice use. Nevertheless, the discovery, dissemination and application of high-quality chemical probes to investigate the function of proteins is expanding. New mechanisms of action have been described and harnessed to achieve greater potency and selectivity. Degraders have shown that it is possible to modulate protein levels in living cells using small molecules, with selectivity demonstrated by quantitative mass spectrometry proteomics. Moreover, obtaining the 3D structure of the tripartite complex involving an E3 ligase and the protein of interest can improve the selectivity of a promiscuous ligand dramatically. This new class of molecules is rapidly expanding our chemical probe toolbox and deflecting the spotlight to proximity pharmacology.^11^ The development of probes and also clinical drug candidates from this class is one of the most exciting recent developments.

Certainly not as prevalent, inhibitors of PPIs have nevertheless also gained their place on the Olympus of high-quality chemical probes. Venetoclax has shown that it is possible to potently inhibit PPIs, achieve selectivity within the target protein family and translate PPI inhibitors into the clinic. However, other PPIs may be more difficult to target and evaluating the selectivity of PPI chemical probes outside the target protein family could be challenging.

Regardless of the mechanism of action, developing chemical probes that are exquisitely selective for a single target can be arduous, especially within the same protein family – although activity against distantly-related proteins can also be troublesome, hence the need for broad profiling. Therefore, we support the development and application, alongside biological tools, of structurally and/or mechanistically different chemical probes targeting the same protein to reduce the risk of being misled by off-target-based conclusions. Inactive analogues provide additional important controls when evaluating the selectivity of chemical probes and the function of proteins. However, they should also be profiled extensively and in parallel with their corresponding active molecule to confirm a reasonable overlap between off-target activity spectra.

**Fig. 3:**
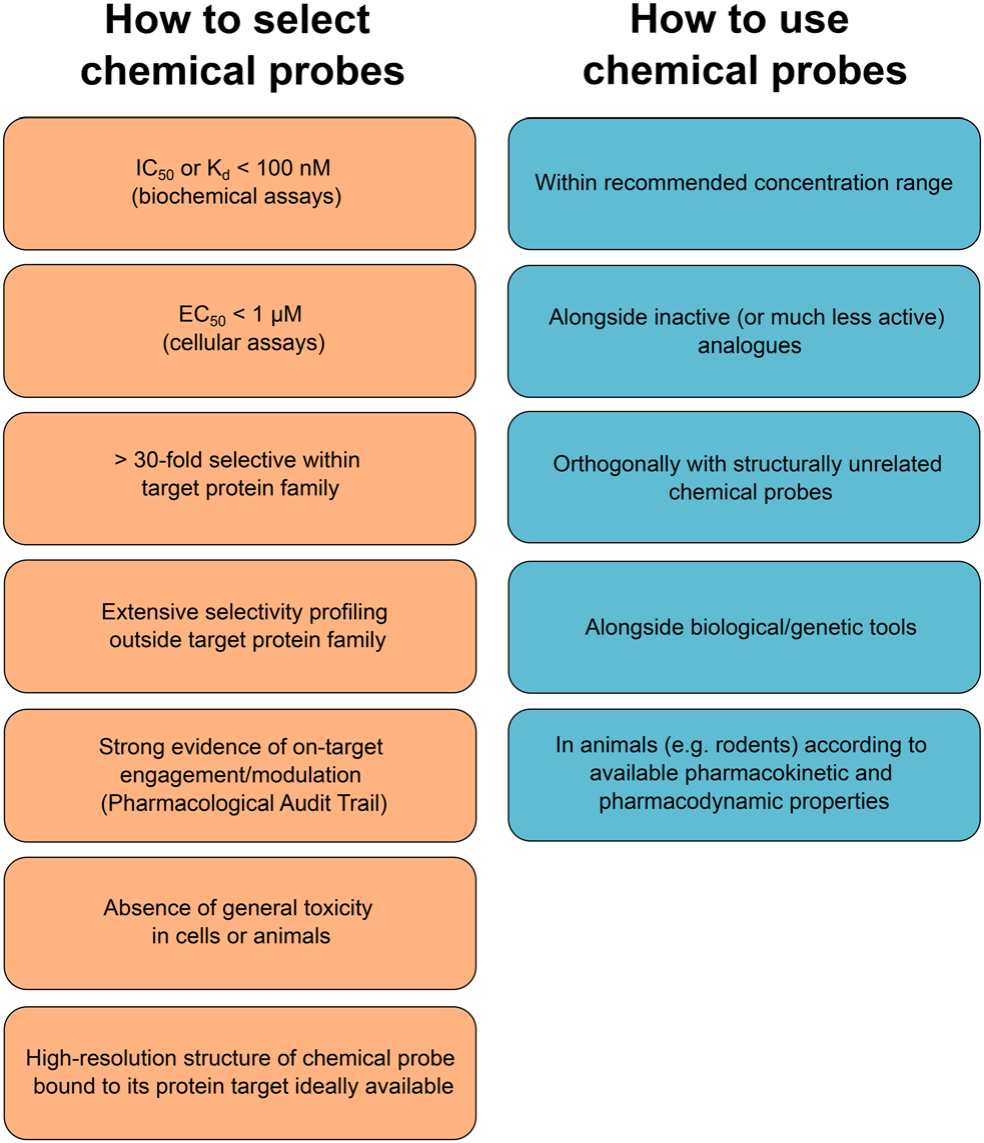
Criteria for the selection and use of chemical probes.

Many new chemical probes can now be obtained freely through different non-profit platforms promoting unencumbered use and further research. However, commercial vendors will continue to play an important role in the wider adoption of high-quality chemical probes in biological research as these are often the only small-molecule providers that biologists seeking chemical probes can turn to. We encourage all commercial vendors to adopt a more transparent approach when labeling small molecules as chemical probes. Experimental data on potency and selectivity should always be disclosed and evaluated in light of the criteria we have outlined above. Various resources have been created to provide chemical probe users with appropriate information to make an informed selection.^38^ The expert-curated database Chemical Probes Portal^19,39–41^ should be referenced when pertinent. To further help chemical probe users with the selection of the most appropriate molecule to study their target of interest, the complementary web-based systematic and objective resource Probe Miner has been created.^42^ These relatively recent developments will certainly accelerate the widespread adoption of high-quality chemical probes and the sanitation of erroneous conclusions inferred upon use of historical poor-quality compounds. Journal editors, grant-funders and reviewers can also provide an important gate-keeper role to promote the selection of high-quality chemical probes and best-practice use (Fig. 3).

Despite recent successes and overall progress, we are still lacking essential chemical tools to study the function of most human proteins. According to a recent estimate, only 11% of the human proteome has been liganded and only 795 human proteins can be investigated using small molecules satisfying minimal potency and selectivity criteria.^42^ Recently, the Target 2035 initiative has set the ambitious goal to create unencumbered chemogenomics libraries, high-quality chemical probes, and/or functional antibodies targeting every human protein.^28,40,90^ With an average estimate of more than $2 million to develop a small-molecule probe, the final objective of Target 2035 will be expensive. Collaborative interactions between academia and industry as well as creative funding mechanisms will be essential. Additionally, a single high-quality ‘conventional’ chemical probe may not be sufficient to fully and robustly capture the detailed function of a protein and a more sophisticated chemical toolbox comprising inhibitors, activators, allosteric modulators and degraders may often be required. However, a more immediate goal of Target 2035 is to galvanize a concerted and focused worldwide research effort towards the development of high-quality chemical probes for selected protein families. This will be empowered by recent advances in synthetic chemistry;^91,92^ DNA-encoded libraries;^93^ experimental protein structural studies, including cryo-EM^94^ and protein structural predictions;^95,96^ biochemical, fragment-based and computational screening;^97,98^ phenotypic screening;^84,99,100^ target engagement technology^101^ and selectivity assays; artificial intelligence;^102^ and other emerging technologies. Moreover, new knowledge on understudied proteins is being generated by initiatives such as the NIH's Illuminating the Druggable Genome,^103^ which focuses on GPCRs, kinases and ion channels that are yet to be targeted, and the RESOLUTE consortium,^104^ which is pioneering research on solute carriers. These large-scale efforts will certainly provide invaluable information for the discovery of high-quality chemical probes targeting new proteins. Inevitably, some proteins will be technically challenging or even impossible to target. Nevertheless, a combined effort over the next 15 years will likely result in a considerable number of significant achievements, a dramatic expansion of our chemical toolbox to investigate protein function and validate drug targets, and the consolidation of the era of high-quality chemical probes.

It should be emphasised that in addition to pushing forward with expanding the proteome coverage and availability of high-quality chemical probes, a parallel concerted outreach effort is required to increase the awareness of the biomedical research community beyond the experts in chemical biology, medicinal chemistry and drug discovery pharmacology, to include the vast number of scientists who use and publish work with chemical probes across a wide range of fundamental and translational research areas. The dissemination of information on criteria for high-quality chemical probes and advice on best practice use (Fig. 3) is important and would benefit from publication in the broader biomedical literature rather than only in specialist journals.^8,24,39–41^ Web resources^38^ including webinars such as those run by the Target 2035 initiative and the Chemical Probes Portal (https://www.chemicalprobes.org/information-centre#presentations), dedicated sessions at relevant conferences, blogs (*e.g.*https://www.icr.ac.uk/our-research/about-our-research/research-themes/chemical-probes) and social media (*e.g.* @Chemical_probes, @target2035) are also crucially important. Learned and professional societies, journals,^39^ funding bodies and vendors can also help promote standards. It is through the combination of greatly expanding the proteome-wide coverage with a much wider awareness of best practice selection and use across biomedical research that the era of high-quality chemical probes will realise its full potential. We encourage participation in these community-led activities.

## Conflicts of interest

M. P. Licciardello and P. Workman are current or past employees of The Institute of Cancer Research which operates a reward to discoverers policy. M. P. Licciardello is now an employee of Relation Therapeutics. P. Workman is a former employee of AstraZeneca. P. Workman is an independent Board Director at Storm Therapeutics; has a consultant/advisory board member role at Alterome Therapeutics, Astex Pharmaceuticals, Black Diamond Therapeutics, CHARM Therapeutics, CV6 Therapeutics, Epicombi Therapeutics, Merck KGaA, Nextechinvest, Storm Therapeutics and Vividion Therapeutics; reports receiving a commercial research grant from Astex Pharmaceuticals, Cyclacel Pharmaceuticals, Merck KGaA, Nuvectis Pharma, Sixth Element Capital and Vernalis; and has ownership interest in Alterome Therapeutics, CHARM Therapeutics, Chroma Therapeutics, Epicombi Therapeutics, Nextechinvest and Storm Therapeutics. P. Workman also serves as Executive Director of the non-profit Chemical Probes Portal.

## Supplementary Material
